# Use of mRNA-seq to discriminate contributions to the transcriptome from the constituent genomes of the polyploid crop species *Brassica napus*

**DOI:** 10.1186/1471-2164-13-247

**Published:** 2012-06-15

**Authors:** Janet Higgins, Andreas Magusin, Martin Trick, Fiona Fraser, Ian Bancroft

**Affiliations:** 1 John Innes Centre, Norwich Research Park, Norwich, NR4 7UH, UK; 2 The Genome Analysis Centre, Norwich Research Park, Norwich, NR4 7UH, UK

## Abstract

**Background:**

Polyploidy often results in considerable changes in gene expression, both immediately and over evolutionary time. New phenotypes often arise with polyploid formation and may contribute to the fitness of polyploids in nature or their selection for use in agriculture. Oilseed rape (*Brassica napus*) is widely used to study the process of polyploidy both in artificially resynthesised and natural forms. mRNA-Seq, a recently developed approach to transcriptome profiling using deep-sequencing technologies is an alternative to microarrays for the study of gene expression in a polyploid.

**Results:**

Illumina mRNA-Seq is comparable to microarray analysis for transcript quantification but has increased sensitivity and, very importantly, the potential to distinguish between homoeologous genes in polyploids. Using a novel curing process, we adapted a reference sequence that was a consensus derived from ESTs from both *Brassica* A and C genomes to one containing separate A and C genome versions for each of the 94,558 original unigenes. We aligned reads from *B. napus* to this cured reference, finding 38% more reads mapping from resynthesised lines and 28% more reads mapping from natural lines. Where the A and C versions differed at single nucleotide positions, termed inter-homoeologue polymorphisms (IHPs), we were able to apportion expression in the polyploid between the A and C genome homoeologues. 43,761 unigenes contained at least one IHP, with a mean frequency of 10.5 per kb unigene sequence. 6,350 of the unigenes with IHPs were differentially expressed between homoeologous gene pairs in resynthesised *B. napus*. 3,212 unigenes showed a similar pattern of differential expression across a range of natural *B. napus* crop varieties and, of these, 995 were in common with resynthesised *B. napus.* Functional classification showed over-representation in gene ontology categories not associated with dosage-sensitivity.

**Conclusion:**

mRNA-Seq is the method of choice for measuring transcript abundance in polyploids due to its ability to measure the contributions of homoeologues to gene expression. The identification of large numbers of differentially expressed genes in both a newly resynthesised polyploid and natural *B. napus* confirms that there are both immediate and long-term alterations in the expression of homoeologous gene pairs following polyploidy.

## Background

Polyploidy or whole genome duplication (WGD) has occurred multiple times throughout the evolutionary history of plants. It has long been recognized as a major force in angiosperm evolution, plant speciation and diversification [1-3]. Polyploidization is both an ancient and an ongoing evolutionary process [4,5] and has played a role in the adaptation of a wide range of crops to different environments by generating phenotypic variation. Polyploids are generally divided into two categories; autopolyploids from duplication of the same genome; and allopolyploids from hybridization of two diverged genomes with subsequent genome duplication. These distinctions are less clear in paleopolyploids. Soybean [6] and maize [7] are considered to be paleopolyploids having been formed between 10 – 15 Mya. Both show evidence of diploidization, an ongoing process by which a newly formed polyploid becomes stabilized, involving the loss of duplicated genes, thereby returning the genome to a diploid-like form [8]. Both potato and alfalfa are derived through autopolyploidy, while wheat, oat, cotton, coffee and oilseed rape have allopolyploidy in their evolutionary history. *Trapogoyon* is a young allopolyploid species that has formed multiple times over the last 80 years [9] and so offers the opportunity to study a natural allopolyploid which is sympatric with its parental species [10]. The success of newly formed angiosperm polyploids is partly attributable to their highly plastic genome structure. Recent studies have documented rapid and dynamic changes in genomic structure and gene expression in plant polyploids. Much of the functional plasticity in polyploids is correlated with gene expression changes at transcriptional and post-transcriptional levels. Such gene expression changes are controlled largely by epigenetic mechanisms [1,2,11].

The *Brassica* species include an important group of vegetable and oil crops and their genomes have complex evolutionary histories. A major focus for research has been *Brassica napus* (oilseed rape). This is an allopolyploid species formed by the hybridization of progenitor species *Brassica rapa* (which contributed the A genome) and *Brassica oleracea* (which contributed the C genome). The *Brassica* species in general, and *B. napus* in particular, provide an excellent system in which to study the impacts of polyploidy and the processes by which genomes subsequently stabilize. *B. rapa* and *B. oleracea* are closely related, having diverged around 3.5 Mya [12]. The *B. napus* types cultivated as crops arose from natural polyploid formation, probably during human cultivation, i.e. less than 10,000 years ago. Genetic mapping studies confirmed that the progenitor A and C genomes are essentially intact in natural lines of *B. napus* and have not been substantially rearranged [13]. It is also possible to make newly constructed (“resynthesised”) polyploids in the laboratory by crossing *B. rapa* and *B. oleracea* accessions and doubling chromosomes (typically by chemical treatment). Song *et al.*[14] used resynthesised polyploids to study genome evolution in the early generations after polyploidization and demonstrated that polyploid species can generate extensive genetic diversity in a short period of time. Pires *et al.*[15] were interested in the ability of polyploids to possess novel traits that are not present in their diploid progenitors which has allowed polyploids to successfully enter new ecological niches. Focussing on flowering time they showed evidence of chromosomal rearrangements and changes in gene expression, which partially explained the phenotypic variation in *B. napus*. The mechanisms for chromosome stability and diploidization in polyploids remain largely unknown but a study of 50 resynthesised lines of *B. napus* showed that in the first generation (S 0 ) of resynthesised *B. napus*, genetic changes are rare but cytosine methylation changes are frequent, whereas in later generations (S 5 ) genetic changes are much more frequent, but the S 0 methylation remained fixed [16]. The genetic changes observed in resynthesised *B. napus* are not random and there is evidence that many are the consequence of homoeologous recombination [17]. Recent cytological investigations including a S 10:11 generation showed that changes in copy number of individual chromosomes increased with successive generations; they showed gross chromosomal rearrangements and that dosage balance mechanisms enforced chromosome number stability [18]. There is much interest on how these genetic and epigenetic changes contribute to changes in gene expression. Transcriptional changes are likely to be a critical component of polyploid evolution as they can contribute directly to novel phenotypes. Most studies have compared gene expression in resynthesised polyploid lines to expression in their parents to provide evidence of additive or non-additive gene expression. According to the "additivity hypothesis”, newly-synthesized allopolyploids are supposed to display mid-parental expression patterns. Many exceptions are found in resynthesised allopolyploids e.g. *Arabidopsis*[19], *Senecio*[20], *Brassica*[17], *Triticum*[21], and *Gossypium*[22], suggesting that the differential regulation of gene expression is a common feature of plant allopolyploids. Although the phenomenon of non-additive expression in inter-specific hybrids and allopolyploids is now well described, the underlying mechanisms are still poorly understood. Recent studies have used statistical methods to predict the contribution of each parent to gene expression in the polyploid using genome-wide microarrays that are not able to distinguish between expression of homoeologous pairs [17,22]. The “additivity” hypothesis was confirmed using comparative proteomics on newly resynthesised *B. napus*[23]. Identification using mass spectrometry and functional categorisation of the differentially regulated proteins did not show that any functional category, metabolic pathway or subcellular localization was over- or under represented within non-additive polypeptides [24]. Comparing transcript levels in resynthesised *B. napus* to protein levels showed that differential protein regulation is not explained by transcriptional changes [25]. This is a complex process so another approach has been to measure transcript levels of homoeologous pairs of genes, but not transcriptome-wide. For example, Dong *et al.*[26] showed a complex pattern of differential expression in response to abiotic stress in both natural and resynthesised allopolyploid *Gossypium hirsutum* using SSCP-cDNA gels to distinguish homoeologous pairs of 60 genes. Also, Chaudhary *et al.*[27] used a mass-spectrometry-based SNP detection technique to measure allele- and homoeologue-specific contributions to the transcriptome of diploid and allopolyploid cotton and showed that 40% of homoeologues were transcriptionally biased in at least one stage of cotton development. Development of a method to measure genome-wide differential expression of homoeologous pairs using transcriptome sequencing in both synthetic and natural polyploids would contribute to our understanding of this complex process.

Next generation sequencing technologies (NGS) have opened exciting opportunities to study genomes and transcriptomes of plant species with and without sequenced genomes. Many crop genome projects are ongoing, including oilseed rape, bread wheat and banana, but many of these polyploid plants have complex genome structures meaning that producing a draft sequence is challenging [28,29]. Meanwhile plant transcriptomics using NGS can yield much information on crops [30], including gene discovery, transcript quantification, post-transcriptional regulation and linking genotypes to phenotypes [31]. mRNA-Seq is a recently developed approach to transcriptome profiling that uses deep-sequencing technologies [32]. Previous experiments, using mRNA-Seq for SNP detection in *B. napus*[33,34], have proved Illumina sequencing to be an efficient method. mRNA-Seq can also be used as a method to estimate transcript abundance. The first step is to map the reads to the genome or transcriptome, and then the number of reads aligning to a specific region of the reference sequence is counted and subjected to relevant normalisation procedures [35]. It is anticipated that mRNA-Seq will revolutionize the manner in which eukaryotic transcriptomes are analysed [32] as sequencing-based approaches have clear advantages over hybridization-based approaches for quantifying the transcriptome. A range of studies comparing microarray and mRNA-Seq have consistently shown that sequencing has higher sensitivity and dynamic range [36,37], although reproducibility has been shown to depend on the type of sample studied [36,38,39] and a recent study has shown that technical variability is too high to be ignored [40].

We previously developed a set of 94,558 *Brassica* unigenes, by assembly of all available *Brassica* ESTs, and used this set for the design of a *Brassica* microarray [41]. We used this for the analysis of gene expression in resynthesised *B. napus* lines (“*B. napus* 1” and “*B. napus* 2”). These resynthesised *B. napus* lines shared the same parental combinations of *B. rapa* (R-o-18) and *B. oleracea* (A12DH), but from reciprocal crosses. The *Brassica* unigenes were assembled using parameters that enabled the separate assembly of transcripts of paralogous genes within each diploid *Brassica* genome (as these differ by ~15% at the nucleotide level) but the co-assembly of transcripts of homoeologous genes (which differ by only ~3%). The surface-bound oligonucleotide probes of the microarrays were designed to regions that differed most between unigenes (typically 3’ untranslated regions), so discriminate well between unigenes representing paralogous genes, but they have no capability to discriminate between homoeologous genes.

In the present study, we report the development of methodology for using mRNA-Seq to quantify transcript abundance in polyploids, with estimation of the relative contributions of homoeologous genes. As a proof of concept, we analysed mRNA from reserved aliquots of the same ground leaf samples taken from the resynthesised *B. napus* plants used in our 2009 microarray-based study. We show, *inter alia*, that mRNA-Seq can be used successfully for both qualitative and quantitative analyses of gene expression and for the apportioning of transcript abundance to A and C genomes in both resynthesised and natural *B. napus*.

## Results and discussion

### Comparison of microarray and mRNA-seq for transcript quantification

**Figure 1:**
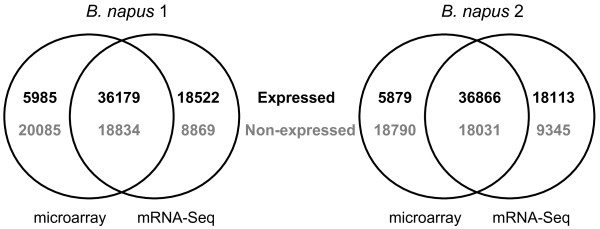
**Qualitative gene expression in resynthesised*B. napus*as determined by microarrays and mRNA-Seq.** Venn diagram showing the numbers of unigenes called as expressed (black) or non-expressed (grey) consistently across all four replicates.

For the first stage of the evaluation of mRNA-Seq, we used the sequences of the original 94,558 *Brassica* unigenes as the reference sequence (hereafter termed the “naive reference”) for the alignment of Illumina mRNA-Seq reads using MAQ [42] . An average of 29.6 million raw 80 base reads was generated per sample. These sequences were of high quality: the mean Phred-like quality value at base 80 was Q27.9 and the mean base position at which it declined below Q30 was 71.6. Across the four biological replicates of *B. napus* 1, an average of 12.3 million reads (0.98 Gb) were mapped to an average of 60,627 unigenes in the aligned naive reference with a mean depth of coverage of 35.3-fold. Similarly for *B. napus* 2, an average of 11.9 million reads (0.96 Gb) were mapped to an average of 60,872 unigenes with mean depth of 34.2-fold. The details are shown in Table 1. Qualitative analyses of gene expression were conducted for both *B. napus* 1 and *B. napus* 2. Unigenes were called as expressed if they had one or more reads aligning in all four replicates and were called as non-expressed if they had zero counts in all four replicates (the remaining unigenes were considered to be inconsistent between replicates). 54,701 and 54,979 unigenes were called as expressed for *B. napus* 1 and *B. napus* 2 respectively, and 27,703 and 27,376 unigenes were called as non-expressed. These results were compared with the calling of unigenes as expressed or non-expressed using the microarray [41]. A comparison of the complements of classified unigenes is shown in Figure 1. The largest class of unigenes is that for which expression has been called by both microarray and mRNA-Seq. Although some unigenes were called as expressed by microarray only, more than three times as many were called as expressed by mRNA-Seq only. This observed greater sensitivity of mRNA-Seq is consistent with previous reports [32] and has been attributed to the very low, if any, background signal present in mRNA-Seq, enabling detection of low levels of expression. Our observation of extensive overlap between genes called as expressed by both microarray and mRNA-Seq is also consistent with the results of studies in other species [43].

**Table 1:** Summary of read alignments

				**Naive reference**					**Cured reference**							
**Sample**	**Experiment**	**Type**	**No. sequence reads**	**No. sequence reads mapped to reference**	**Aligned reference sequence** **/bases**	**Average depth over aligned reference**	**No. unigenes with mapped sequence reads**	**Total sequence mapped to naive reference/Gb**	**No. sequence reads mapped to A genome**	**No. A genome unigenes with mapped sequence reads**	**No. sequence reads mapped to C genome**	**No. C genome unigenes with mapped sequence reads**	**Aligned reference sequence** **/bases**	**Average depth over aligned reference**	**Total sequence mapped to cured reference/ Gb**	**Increase sequence mapped to reference** **/ %**
*B. napus* 1 rep1	BCC_1_2*	Resynthesized (*B. rapa* cytoplasm)	30290727	11396746	27704492	32.9	60801	0.91	8136121	57101	7725545	57232	48716080	26.0	1.27	39.2
*B. napus* 1 rep2	BCC_2_2_rep*		32362747	12596459	27628537	36.4	60182	1.01	8974130	56758	8472600	56859	48927782	28.5	1.40	38.5
*B. napus* 1 rep3	BCC_3_2*		30212318	12688970	28367947	35.8	60953	1.02	8933134	57381	8523534	57505	50115239	27.9	1.40	37.6
*B. napus* 1 rep4	BCC_4_2*		29073842	12556527	27816387	36.1	60572	1.00	8886730	57072	8581997	57168	49020495	28.5	1.40	39.1
*B. napus* 2 rep1	BCC_1_4*	Resynthesized (*B. oleracea* cytoplasm)	28333122	11390102	28050282	32.5	61139	0.91	8138583	57406	7545728	57600	49388101	25.4	1.25	37.7
*B. napus* 2 rep2	BCC_2_4*		27156815	11209306	27415043	32.7	59994	0.90	7908682	56465	7619986	56551	48167524	25.8	1.24	38.5
*B. napus* 2 rep3	BCC_3_4*		29720775	12618159	28214425	35.8	61051	1.01	8828851	57410	8491265	57632	49691719	27.9	1.39	37.3
*B. napus* 2 rep4	BCC_4_4*		29830870	12580532	28294428	35.6	61303	1.01	8829979	57645	8473127	57779	49861276	27.8	1.38	37.5
Tapidor	TAP**	Winter	22841910	11026783	28473370	31.0	61776	0.88	6770387	57940	7281348	58231	48844912	23.0	1.12	27.4
Ningyou 7	NIN**	Chinese	23151184	10774087	28233727	30.5	61982	0.86	6640976	58055	6841509	58167	48266879	22.3	1.08	25.1
Altasweet	D3_ALT*	Swede	29080184	13239756	28730927	36.9	61251	1.06	8340513	58022	8806044	58177	49777567	27.6	1.37	29.5
Ceska	D31_CES*	Spring	28036702	13193597	29567877	35.7	61903	1.06	8050594	58512	8803320	58830	51209098	26.3	1.35	27.7
Aphid Resistant Rape	D8_AphRR*	Fodder	28334337	11879400	29161922	32.6	62434	0.95	7486610	58922	7881247	59083	50269176	24.5	1.23	29.4

* deposited under SRA accession ERA063602.

**deposited under SRA accession ERA036824.

**Figure 2:**
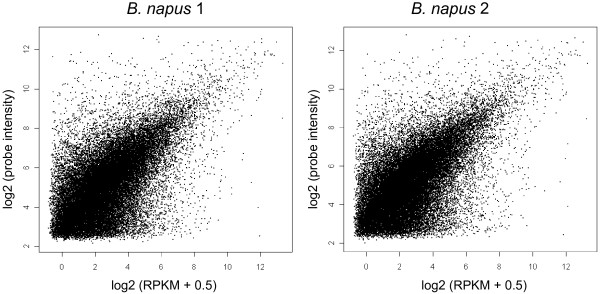
**Comparison of transcript quantification in resynthesised*B. napus*by microarrays and mRNA-Seq.** Mean transcript quantification values across four biological replicates are plotted as RPKM values for mRNA-Seq against probe intensities for microarray expression levels.

For the second stage of the evaluation, we compared transcript quantification by microarrays and mRNA-Seq. For the unigenes called as expressed by both mRNA-Seq and microarrays (36,179 for *B. napus* 1 and 36,866 for *B. napus* 2), we quantified and normalized transcript abundance (as reads per kb per million aligned reads (RPKM) for mRNA-Seq, absolute intensities for microarray). These showed good correlation for both *B. napus* 1 and *B. napus* 2 (Pearson correlation = 0.59), as shown in Figure 2. This degree of correlation is consistent with the results of previous studies using two-colour Agilent microarrays [44]. Our analysis was conducted across the entire range of measured transcript abundance (0.02 – 27,000 RPKM) demonstrating that good correlation can be detected beyond just the medium abundance range shown previously [32]. The combination of the wider dynamic range of mRNA-Seq, in addition to its previously reported ability to provide a better estimate of absolute expression levels [45], confirms mRNA-Seq as the preferred methodology for transcript quantification.

### Modifying the reference sequence to improve read alignment

**Figure 3:**
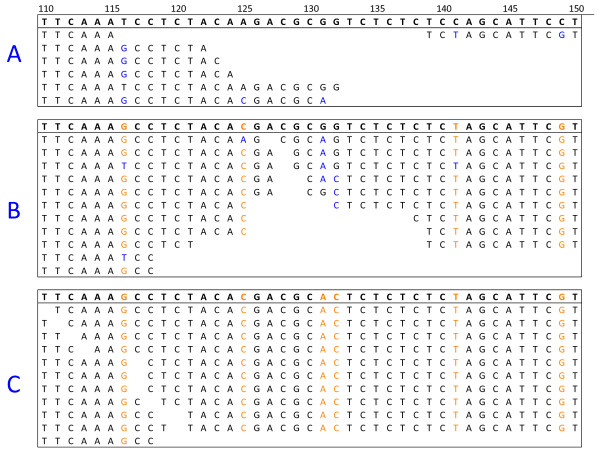
**Example of the A genome curing process for a section of unigene.** Each panel shows an example alignment window for a section of a unigene between bases 110 and 150. Within each panel, the top line is the reference sequence, underneath are the aligned of 40 base *B. rapa* reads. Base differences from the reference are shown in blue. Cured bases in the reference and aligned reads are shown in orange. Panel A shows cycle 1 of the curing process. A small number of *B. rapa* reads are aligned against the naive reference sequences in sections of the unigene where there are no more than 3 mismatches. At the end of the curing process at position 116, the T will be changed to a G in the reference sequence, this will enable more reads to align in this section of the unigene on the next cycle. Panel B shows cycle 3. Changes have been made in the reference sequence for positions 116 (T → G), 125 (A → C), 141 (C → T), 149 (C → G), these are shown in orange. This has enabled more reads to align to the middle section of the unigene. Panel C shows cycle 6. A further two changes have been made in the reference sequence at positions 131 (G → A), 132 (C → G). At this stage, a large number of *B. rapa* reads are aligning against the reference sequence with no mismatches. The reference sequence now becomes the A genome ‘cured’ reference.

**Figure 4:**
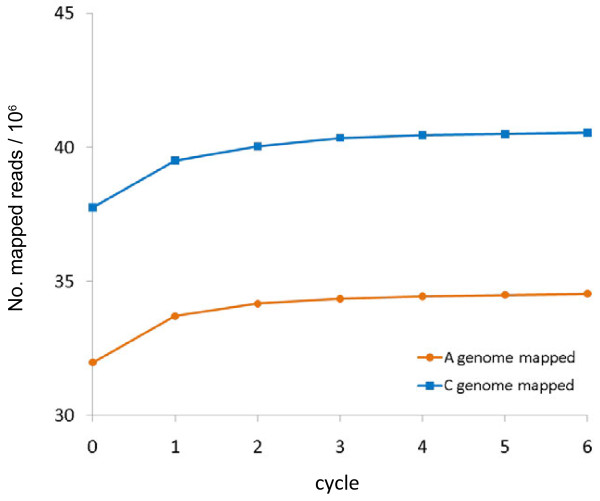
**Increase in number of mapped reads following curing of the naive reference sequence.** Number of mapped reads following each cycle of curing.

**Figure 5:**
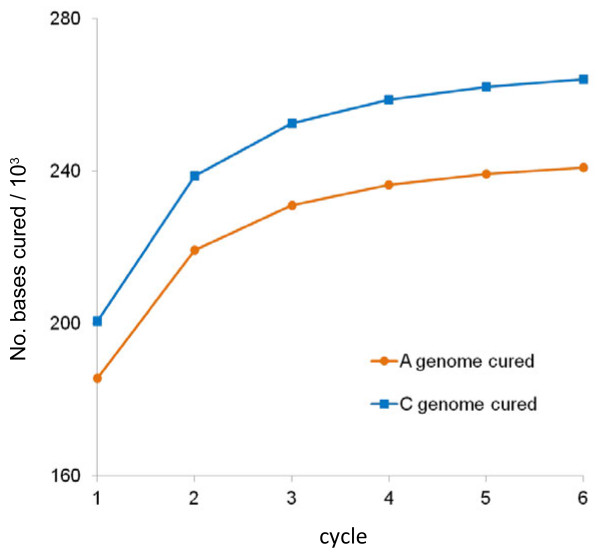
**Curing the unigenes to more closely match sequences from*B. rapa*and*B. oleracea*.** Cumulative number of bases cured over the 6 cycles.

**Figure 6:**
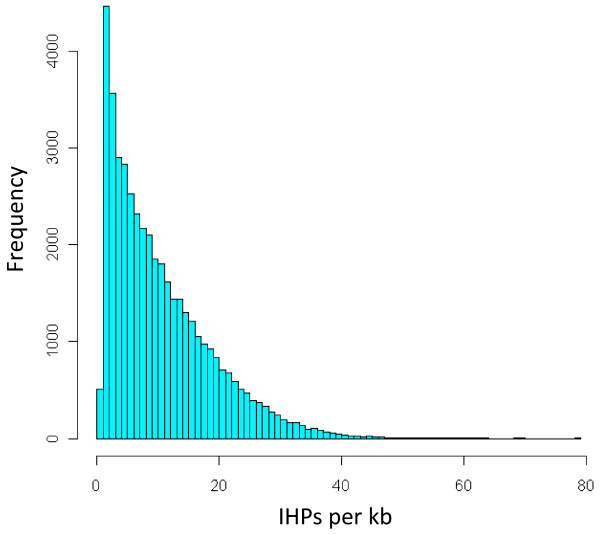
**Density of inter-homoeologue polymorphisms detected.** Frequency distribution of the density of IHPs detected per kb of unigene sequence for 43,761 unigenes.

The unigene assemblies are derived from sequences originating from multiple cultivars and even multiple genomes, so may differ from any actual transcript sequences. If the number of mismatches between an individual Illumina read and the most similar unigene in the naive reference sequence is too great, it will not map. In order to increase the proportion of sequence reads that map, the reference sequence can be corrected, to more closely represent the two genomes present in the polyploid. In the case of *B. napus*, the improved reference sequence, which we term “cured” can be produced by separately curing the naive reference with *B. rapa* mRNA-Seq reads and *B. oleracea* mRNA-Seq reads so that the unigene variants more closely represent transcripts from the A and C genomes respectively. As the curing process is expected to progressively allow the mapping of additional reads, the process should be conducted iteratively over a number of cycles. An initial version of this process has been reported previously [33]. However, we improved upon this by splitting the available 80 base Illumina reads from *B. rapa* and *B. oleracea* to 2 x 40 base reads. This allowed mapping of reads to regions of reference sequence with higher divergence. We applied the curing process iteratively over six cycles, using 46,120,559 reads for *B. rapa* and 49,268,765 reads for *B. oleracea*. The curing process is shown schematically in Figure 3 for the A genome at cycles 1, 3 and 6. The number of reads mapped after each round of curing is shown in Figure 4 and the cumulative number of bases cured is shown in Figure 5. The resulting *B. rapa*-cured and *B. oleracea*-cured reference sequences contained 240,908 and 264,106 base differences from the naive reference, respectively (57,188 base changes were concerted between *B. rapa*-cured and *B. oleracea*-cured reference sequences). Nevertheless, there were 385,789 base differences (excluding unassigned,”N”, base calls), within 43,761 unigenes, between the *B. rapa*-cured (or A genome-cured) and *B. oleracea*-cured (or C genome-cured) unigene sequences. These differences represent inter-homoeologue polymorphisms (IHPs). The number of IHPs per unigene in this subset of 43,761 unigenes ranged from 1 to 81. As the unigenes are of different lengths, the density of IHPs identified in the unigenes was calculated. The frequency distribution is illustrated in Figure 6. Although most unigenes have a low density of IHPs, with the mean being 10.5 IHPs per kb, a few had high frequencies, with a maximum of 78.6 IHPs per kb.

The *B. rapa*-cured and *B. oleracea*-cured unigene sequences were simply combined to produce the cured reference sequence, which thus comprised the A and C genome variants for each unigene (189,116 unigene sequences in all). Sequences (as intact 80 base reads) from the 4 biological replicates of *B. napus* 1 and *B. napus* 2 were re-mapped to the cured reference. The results are shown in Table 1. Starting with an average of 30.5 million raw reads for *B. napus* 1, an average of 8.7 million reads aligned to A genome versions of the unigenes (57,078 unigenes)and an average of 8.3 million reads aligned to the C genome versions of the unigenes (57,191 unigenes), this represented a total number of reads aligning to the cured reference of an average of 17.1 million reads with a mean depth of coverage of the aligned reference of 27.7-fold, which is an increase of an average of 4.7 million reads (38.6%) compared to the naive reference. Similar results were obtained for *B. napus* 2, which showed a 37.8% increase in the number of mapped reads. These results confirmed that the curing process was highly successful in enabling the mapping of a greater proportion of sequence reads from the resynthesised *B. napus*. The *B. rapa*-cured and *B. oleracea*-cured unigene sequences will be of value for exploiting mRNA-Seq for transcript quantification in these species. The large number of IHPs differentiating the unigenes in the cured reference (385,789) also provides the opportunity to estimate the relative contributions to the transcriptome of homoeologues in the A and C genomes.

### Estimation of the relative contributions of homoeologues to the transcriptome

The mapping to the cured reference of sequences from the four biological replicates of *B. napus* 1 and *B. napus* 2 results in the assignment of each sequence read to the unigene with the fewest mismatches. Where MAQ finds equally probable matches, the read is assigned randomly to one of them. Consequently, the cured reference provides the opportunity to estimate the relative contributions to the transcriptome of homoeologues based on this apportioning of reads, but with an anticipated underestimation of differences between the contributions of homoeologues in the A and C genomes where there are tracts of identical sequence in the corresponding unigenes in the cured reference.

**Figure 7:**
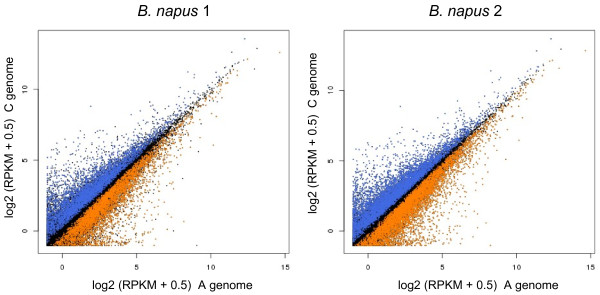
**Apportioning of transcript abundance to A and C genomes in resynthesised*B. napus*.** Mean RPKM values over the four replicates of resynthesised *B. napus* for the A genome version plotted against the C genome version for each of the 43,761 unigenes containing IHPs. Unigenes with significantly (P < 0.05) higher abundance from the A genome copy across the four replicates are plotted in orange; unigenes with significantly (P < 0.05) higher abundance from the C genome copy are plotted in blue.

To assess whether mRNA-Seq could be used to detect quantitative differences between the abundance of transcripts originating from the A and C genomes of resynthesised *B. napus*, we analysed the numbers of reads mapped to each version (A genome-cured and C genome-cured) of the unigenes in the cured reference. Using the separate RPKM values for alignment to the A genome-cured and C genome-cured versions of each unigene as paired values for each of the four replicates of *B. napus* 1 and *B. napus* 2, we were able to identify homoeologous pairs of genes for which the A and C genome versions show significant differences in transcript abundance. Plots of the transcript abundance measured for the A and C genome versions of unigenes are shown in Figure 7 for each of *B. napus* 1 and *B. napus* 2, with unigenes showing significant (P < 0.05) differences between the genome versions highlighted. In all, 6,350 of the 43,761 unigenes containing IHPs showed differential expression (P < 0.05) between the A and C genome homoeologues in both *B. napus* 1 and *B. napus* 2, with 3,179 showing higher expression in the A genome homoeologue and 3,171 showing higher expression in the C genome homoeologue. This set of unigenes was enriched for IHPs (with an average of 14.8 per kb, compared with the overall average of 10.5 per kb). The success of mapping sequence reads to a reference sequence cured to better represent the genomes of ancestral species demonstrates the feasibility of using mRNA-Seq to apportion transcript abundance to the constituent genomes of polyploids, at least for genes in which sufficient IHPs serve to differentiate homoeologues.

To test whether our method for the apportioning of transcript abundance to genomes is also applicable to natural *B. napus*, we generated leaf mRNA-Seq data from five cultivars representing differing crop types. The sequence datasets consisted of between 23 and 29 million raw reads, the mean Phred-like quality value at base 80 was Q26.8 and the mean position at which the quality value declined below Q30 of 60.8. The sequence reads were mapped to both the naive reference and the cured reference. As was observed with mRNA-Seq reads from the resynthesised *B. napus*, more sequences were mapped to the cured reference than to the naive reference, as summarised in Table 1. However, the increase for natural *B. napus* of 27.8%, was lower than that observed for resynthesised *B. napus* (~38%). The difference is likely to be due to the curing process having used sequences from the precise progenitors of the resynthesised *B. napus*, resulting in optimum read mapping, whereas the actual progenitors of the natural *B. napus* types are unknown. We observed consistent (but not significant) biases in the proportions of reads mapping to the A and C genome versions of unigenes for both resynthesised *B. napus* (the majority, 51.2%, mapping to the A genome) and natural *B. napus* (the majority, 51.5%, mapping to the C genome). This may be a consequence of the transcriptome sequence of *B. oleracea* A12DH being more similar to that of the C genome ancestor of natural *B. napus* than the transcriptome sequence of *B. rapa* R-o-18 is to that of the A genome ancestor of natural *B. napus*, which has been observed previously for some regions of the genome [46].

**Figure 8:**
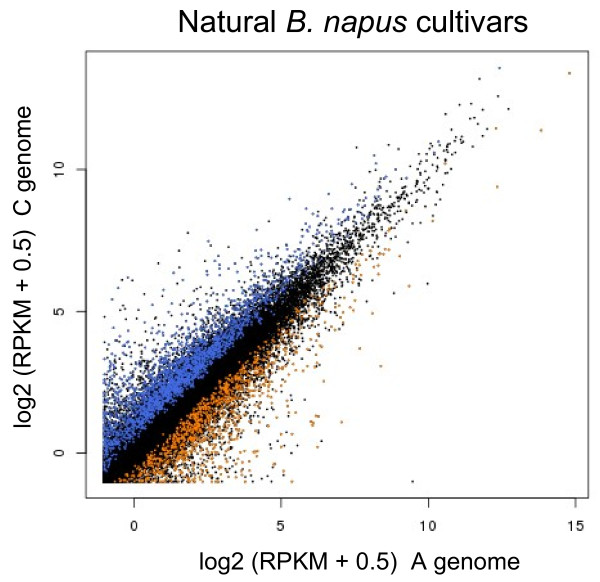
**Apportioning of transcript abundance to A and C genomes in*B. napus*cultivars.** Mean RPKM values over the five varieties of natural *B.napus* for the A genome version plotted against the C genome version for each of the 43,761 unigenes containing IHPs. Unigenes with significantly (P < 0.05) higher abundance from the A genome copy are plotted in orange; unigenes with significantly (P < 0.05) higher abundance from the C genome copy are plotted in blue.

We were also interested in identifying unigenes which were consistently differentially expressed across all the five *B. napus* cultivars. We used the RPKM values for alignment to the A genome-cured and C genome-cured versions of each unigene as paired values to identify homoeologous pairs of genes for which the A and C genome versions showed differences in transcript abundance across all five cultivars. The results are shown in Figure 8, with unigenes showing significant (P < 0.05) differences between the genome versions highlighted. In all, 3,212 unigenes showed differential expression (P < 0.05) between the A and C genome homoeologues, with 1,082 showing higher expression in the A genome homoeologue and 2,130 showing higher expression in the C genome homoeologue. This set of unigenes was enriched for IHPs (with an average of 14.4 per kb, compared with the overall average of 10.5 per kb). This demonstrates the applicability of using mRNA-Seq to apportion transcript abundance to the constituent genomes of natural polyploids.

### Analysis and functional classification of homoeologous unigene pairs showing differential transcript abundance

**Figure 9:**
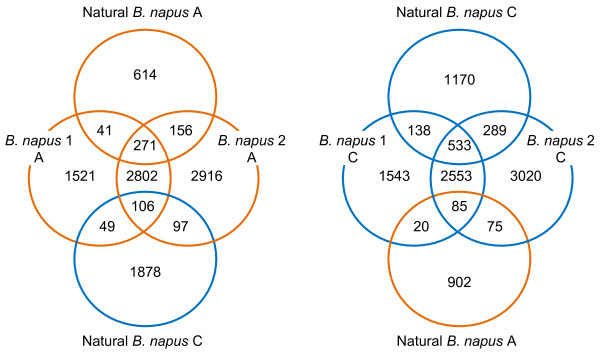
**Unigenes with differential transcript abundance apportioned to A and C genomes in*B. napus*types.** The number of unigenes with higher abundance from the A genome copy is shown in orange circles; the number with higher abundance from the C genome copy is shown in blue circles.

**Figure 10:**
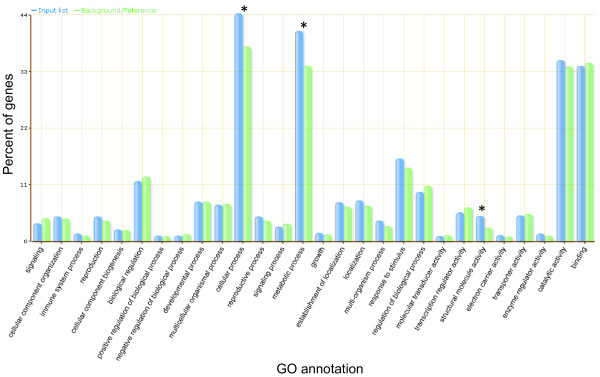
**Functional analysis of genes differentially expressed between homoeologous gene pairs.** Flash bar chart of top level GO terms for biological process and molecular function for the 995 unigenes differentially expressed in both resynthesised and natural *B.napus.* * Over-represented GO terms in these categories (P < 0.05).

In order to assess the consistency between *B. napus* 1, *B. napus* 2 and natural *B. napus* for the inferred differential contributions to the transcriptome from A and C genome homoeologues, we analysed the lists of unigenes identified as differentially expressed and grouped them as shown in Figure 9. There were many instances of differential transcript abundance represented in more than one *B. napus* type. A total of 4,790 unigenes showed higher abundance from the A genome in *B. napus* 1, of these, 3,179 were in common with *B. napus* 2 and 312 showed consistent higher abundance across all five varieties of natural *B. napus*. A similar pattern was observed for unigenes showing higher abundance in the C genome (3,171 in both *B. napus* 1 and *B. napus* 2) except that we found a higher number differentially expressed in natural *B. napus* (671 in both *B. napus* 1 and natural *B. napus*). Focussing on unigenes which were differentially expressed in both resynthesised and natural *B. napus*, we found that 271 unigenes were differentially expressed in both, with higher abundance in the A genome. Interestingly, a further 106 were differentially expressed in both, but showed higher abundance in the C genome of natural *B. napus* giving a total of 377 unigenes. Similarly for unigenes showing higher abundance in the C genome of resynthesised *B. napus*, there were 533 in common with the C genome of natural *B. napus* and 85 in common with the A genome of natural *B. napus* giving a total of 618, thus giving a total of 995 unigenes differentially expressed in all. A functional classification of these 995 differentially expressed unigenes (using corresponding Arabidopsis genes) was carried out using the agriGO web-based Singular Enrichment Analysis (SEA) [47]. Figure 10 shows a comparison of the input list, compared to a background of the 43,761 unigenes that contained IHPs. Significant over-representation was found in GO categories within cellular process and metabolic process. Categories associated with biological regulation such as signalling and transcription regulator activity appear to be under-represented but this is not significant. This functional analysis of unigenes which are differentially expressed shows over-representation of categories which are not significantly over-retained in polyploids [48]. According to the “gene balance hypothesis” [49], genes whose products function in multi-subunit complexes or signalling cascades will tend to be dosage sensitive because changes in the stoichiometry of individual subunits lead to improper assembly and/or function. These dosage sensitive genes include those involved in biological regulation such transcription factors and members of signal transduction pathways, these genes show “non-additive” expression and are typically retained in duplicate in polyploids [11]. Genes encoding products with few interactions have fewer constraints on their expression levels so would be more likely to become differentially expressed between homoeologous gene pairs. This flexibility in their expression levels may contribute to the immediate evolutionary advantage of polyploidy.

## Conclusion

Our investigations have shown that mRNA-Seq represents an excellent approach to the analysis of both qualitative and quantitative transcript abundance in *B. napus*. The increased sensitivity and dynamic range compared with a corresponding analysis using microarrays, along with the good (but far from perfect) correlation of transcript abundance quantification is consistent with the results of previous studies conducted using organisms with simpler genomes. As the cost to sequence a sample is now comparable to the cost to conduct a microarray analysis and software tools are available to manage and mine mRNA-Seq data, we conclude that mRNA-Seq will frequently be the method of choice.

Our studies have shown that mRNA-Seq data can be used to overcome a key problem in polyploid species: the presence of homoeologous genes with very similar sequence being transcribed from each genome. By “curing” the reference unigene sequences so they more closely represent the sequences of the two genomes contained in *B. napus*, we have been able to allocate, on a genome-wide basis, transcript abundance to the individual genomes. Although limited to regions of unigenes in which inter-homoeologue polymorphisms occur, such regions are in the majority and the approach identified many homoeologous transcripts with differential expression from the two genomes. This approach should be generally applicable to polyploid species, so long as diploid representatives of the constituent genomes are available to support the curing process. Moreover, the identification of large numbers of genes with such transcription biases, in replicated experiments, demonstrates that stable homoeo-allelic variation for transcript abundance is common in *B. napus*.

This method also enabled us to successfully study differential expression of homoeologous genes across a range of natural *B. napus* crop varieties. For many genes, the ancestral profiles have been partitioned between the homoeologues in a similar pattern, despite many generations of independent evolution. This is consistent with observations in other polyploids, such as cotton (*Gossypium*) [26,27] and *Spartina*[50], that there are both immediate and long-term alterations in the expression of homoeologous gene pairs following polyploid formation. Functional classification of genes differentially expressed between homoeologous gene pairs showed enrichment for classes not associated with gene dosage effects, consistent with the notion that such gene classes can show allelic variation for transcript abundance that might, itself, represent a class of molecular marker.

## Methods

### Growth of plants and preparation of RNA

The *B. napus* 1 and *B. napus* 2 RNA samples were extracted from stored aliquots of the same ground leaf tissue used for the microarray experiment as described by Trick *et al.*[41]. Five varieties of natural *B. napus* were selected to represent the main crop types: Tapidor, Ningyou 7, Altasweet, Ceska and Aphid Resistant Rape. The plants were grown and the RNA extracted using the same experimental design, growth and harvesting conditions as described for the *B. napus* 1 and *B. napus* 2 plants by Trick *et al.*[41].

### Illumina RNA-sequencing

The sequencing libraries were prepared using the Illumina mRNA-Seq kit (RS-100-0801, Illumina Inc.) as described by Bancroft *et al.*[33]. Each library was run on a single lane for 80 cycles on the Illumina Genome Analyzer GAIIx. Illumina base calling files were processed using GERALD to produce a sequence file containing 80 base reads for each sample in FASTQ format. The Illumina FASTQ format was converted to Sanger FASTQ format before further processing.

### Mapping of reads to the reference sequence and generation of read counts using MAQ

MAQ version 0.7.1 [42] was used to align the 80 base Illumina reads to the 95 k naive reference sequence following the protocols described in the online documentation (http://maq.sourceforge.net) and adopting the default parameter values. MAQ pileup text files were generated from the MAQ binary map files. The Perl script tagcounter.pl (Additional file 1) was used to count the number of reads aligning to each unigene by accessing the pileup files, outputting a count and calculated RPKM value (reads per kb per million aligned reads) for each unigene.

### Comparison of unigene expression measurements obtained using the Agilent microarray with illumina mRNA-seq count data

Data for the four biological replicates of each of *B. napus* 1 and *B. napus* 2 was obtained from the Agilent 60-mer oligonucleotide microarray experiment already published by Trick *et al.*[41] and available from the GEO repository, accession number GSE15915. Unigenes were called expressed, marginally expressed or non-expressed using the Agilent microarray. A similar classification of unigenes was carried out using the Illumina count data. Expressed unigenes were defined as having one or more reads aligning to the unigene in all of the 4 replicates; non-expressed unigenes were defined as having zero counts in each of the 4 replicates, the remaining unigenes were not classified. The statistical programming language R [51] was used to compare the lists obtained from both methods using Set Analysis. Above-background quantitative signal values were obtained for the Agilent microarray. These were correlated with the RPKM values (normalised transcript abundance) obtained using Illumina mRNA-Seq for the unigenes which were called expressed using both methods.

### Preparation of the cured reference sequence

Libraries prepared from *B. rapa* and *B. oleracea* RNA samples were each run on two lanes of the Illumina Genome Analyzer GAIIx for 80 cycles. The FASTQ files from the two lanes were combined generating a total of 46,120,559 reads for *B. rapa* and 49,268,765 reads for *B. oleracea*. The 80 base reads were split into two files, each containing a set of 40 base reads using the Perl script illumina_split_read.pl (Additional file 2). The 40 base reads were used separately to cure the naive reference sequence to an A genome version and a C genome version, described as follows. Using the Perl script cure_cycle_split.pl (Additional file 3), the 40 base reads were aligned against the naive reference sequence to produce a map file. The map files generated by alignment of the first and second sets of 40 base reads were merged using MAQ mapmerge and a consensus sequence generated. The Perl script, cure_refseqs.pl (Additional file 4), was used to cure the naive reference using the consensus sequence. This process was iterated over six cycles after which there was no significant gain in alignment efficiency. On each iteration, bases were replaced in the reference, where these differed from high quality consensus bases called by MAQ (i.e. contributed by a read depth greater than 3, with quality values greater than 40). This process resulted in the production of an A genome and C genome version of the naive reference, these two sequences were compared at each base position using the Perl script compare_sequences.pl (Additional file 5) to give a list of positions within unigenes where the base differed in the two sequences. The cured reference sequence was constructed by combining the two ‘cured’ reference sequences, thus creating a reference sequence containing both the A and C variants of each unigene.

### Mapping of reads to the cured reference and the apportioning of reads to the A and C genome

*B. napus* 1 and *B. napus* 2 samples were re-aligned against the cured reference using MAQ. When a read maps equally well to multiple positions, MAQ will randomly pick one position, thereby distributing reads evenly between the A and C genome versions of the unigene where the sequence is identical. Using the pileup files, the Perl script ACtagcounter.pl (Additional file 6) was used to generate a count of the number of reads and corresponding RPKM value, separately for the A and C genome version of each unigene.

RPKM values for the number of reads aligning to the A and C genome versions of each unigene were analysed in R. Following the principle of *Occam’s razor*, the simplest distribution, Poisson, was fitted to the paired RPKM values across the four replicates of each cross using a Generalised Linear Model (GLM); with the paired structure of the data captured algebraically in the design matrix Chi. However, evident over-dispersion in the data necessitated the use of a quasi-Poisson link function. That is, the variance and mean regression functions can be obtained from a Poisson GLM [52] but the dispersion parameter, Phi, is not fixed at 1 but left unrestricted and subsequently estimated from the data. Hence, we obtain the same estimates of coefficients as for a standard Poisson, but inference becomes effectively over-dispersion-adjusted. The resulting *p*-values were subjected to Benjamini-Hochberg adjustment for multiple testing; and we regarded adjusted *p*-values < 0.05 as significant. Using the same statistical analysis, we compared the five *B. napus* cultivars with the aim of identifying unigenes which were consistently differentially expressed across all five varieties.

### Identifying biological functions of differentially expressed genes

BLASTN hits in Arabidopsis (p ≤ 1.0E-30) were found for 62,383 of the 94,558 brassica unigenes (90,864 unique sequences [41]). The top Arabidopsis hit corresponding to each *Brassica* unigene was used for functional analysis. Functional classification was carried out using the agriGO web-based GO analysis toolkit [47]. Arabidopsis genes (corresponding to each unigenes) were input into the Singular Enrichment Analysis (SEA) using the 43,761 unigenes with IHPs as a customised reference background (15,173 corresponding Arabidopsis genes). The Fisher’s Exact Test with Bonferroni-adjusted *p*-values was employed in the SEA analysis using the “complete GO” ontology.

### Accession codes

Short read sequence data have been deposited at the Sequence Read Archive (SRA) under accession number ERA063602, except for Tapidor and Ningyou 7, which were deposited previously under accession number ERA036824.

## Competing interests

The authors declare that they have no competing financial interests.

## Authors' contributions

I.B, M.T and JH conceived and planned the project. F.F. prepared the libraries. J.H., A.M. and M.T. performed data analysis. J.H., I.B., A.M. and M.T. wrote the manuscript and all authors reviewed it. All authors read and approved the final manuscript.

## Supplementary Material

Additional file 1 Perl program (tagcounter.pl) to parse a MAQ “pileup” file and count the number of reads mapping to each unigene, outputting a read count and calculated RPKM. Click here for file

Additional file 2 Perl program (Illumina_split_read.pl) to split 80 base Illumina FASTQ reads into 2 files containing the first 40 base and last 40 base of each read. Click here for file

Additional file 3 Perl program (cure_cycle_split.pl) calling MAQ to align reads to a reference, then calling cure_refseqs.pl to perform curing of the reference sequence over a specified number of cycles. Click here for file

Additional file 4 Perl program (cure_refseqs.pl) to “cure” the naive reference using the consensus sequence obtained from alignment of reads from target genome. Click here for file

Additional file 5 Perl program (compare_sequences.pl) to compare two sequences and output a list of positions at which base differences occur together with the base call. Click here for file

Additional file 6 Perl program (ACtagcounter.pl) to parse and Illumina “pileup” file and count reads for the A and C version of each unigene, outputting a read count and calculated RPKM. Click here for file
